# Supplementary material for: Identification of EGF Receptor and Thrombospondin-1 as Endogenous Targets of ER-Associated Degradation Enhancer EDEM1 in HeLa Cells
Source: Int J Mol Sci. 2023 Jul 29;24(15):12171. doi: 10.3390/ijms241512171 (PMC10418772; doi:10.3390/ijms241512171)
Supplement: Supplementary file 1 [file ijms-24-12171-s001.zip › Figures S1-S4.docx]

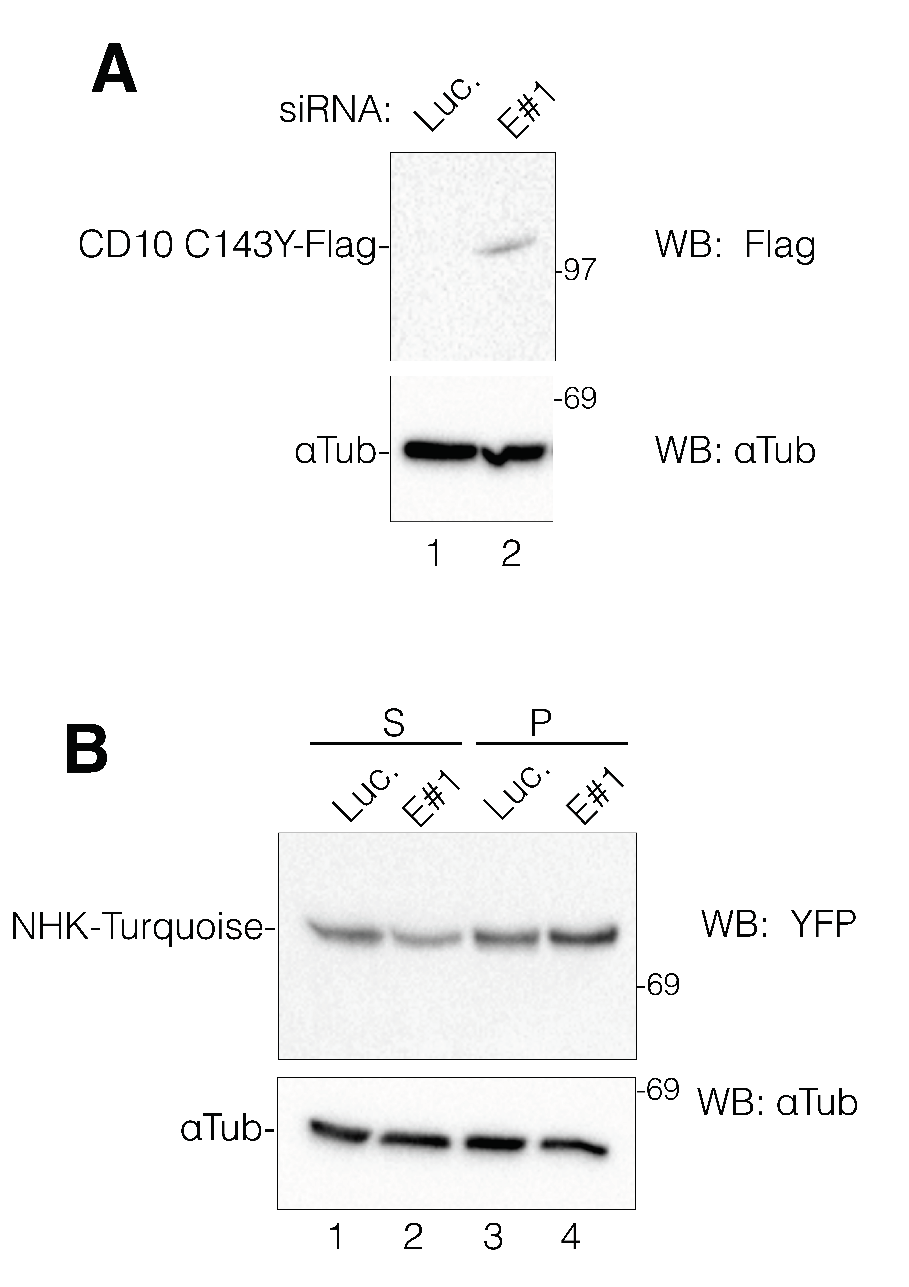


**Figure S1. Knockdown of EDEM1 suppressed ERAD of misfolded proteins in HeLa cells**

Examination of ERAD after treatment of HeLa cells with siRNA against EDEM1. (**A**) HeLa cells were treated with siRNA for negative control (luciferase, Luc.) or EDEM1#1 for 24 h and subsequently transfected with CD10 C143Y-Flag for 48 h (total duration of siRNA treatment: 72 h). Cell lysates were resolved by western blotting using anti-Flag (for CD10 C143Y) or anti-αTubulin antibodies. (**B**) HeLa cells were treated with siRNA for negative control (luciferase, Luc.) or EDEM1#1 for 24 h, and transfected with NHK-Turquoise for 48 h (total duration of siRNA treatment: 72 h). Cell lysates were fractionated into supernatant (S) and precipitation (P) as detergent-insoluble proteins. NHK-Turquoise and αTubulin were detected with indicated antibodies.


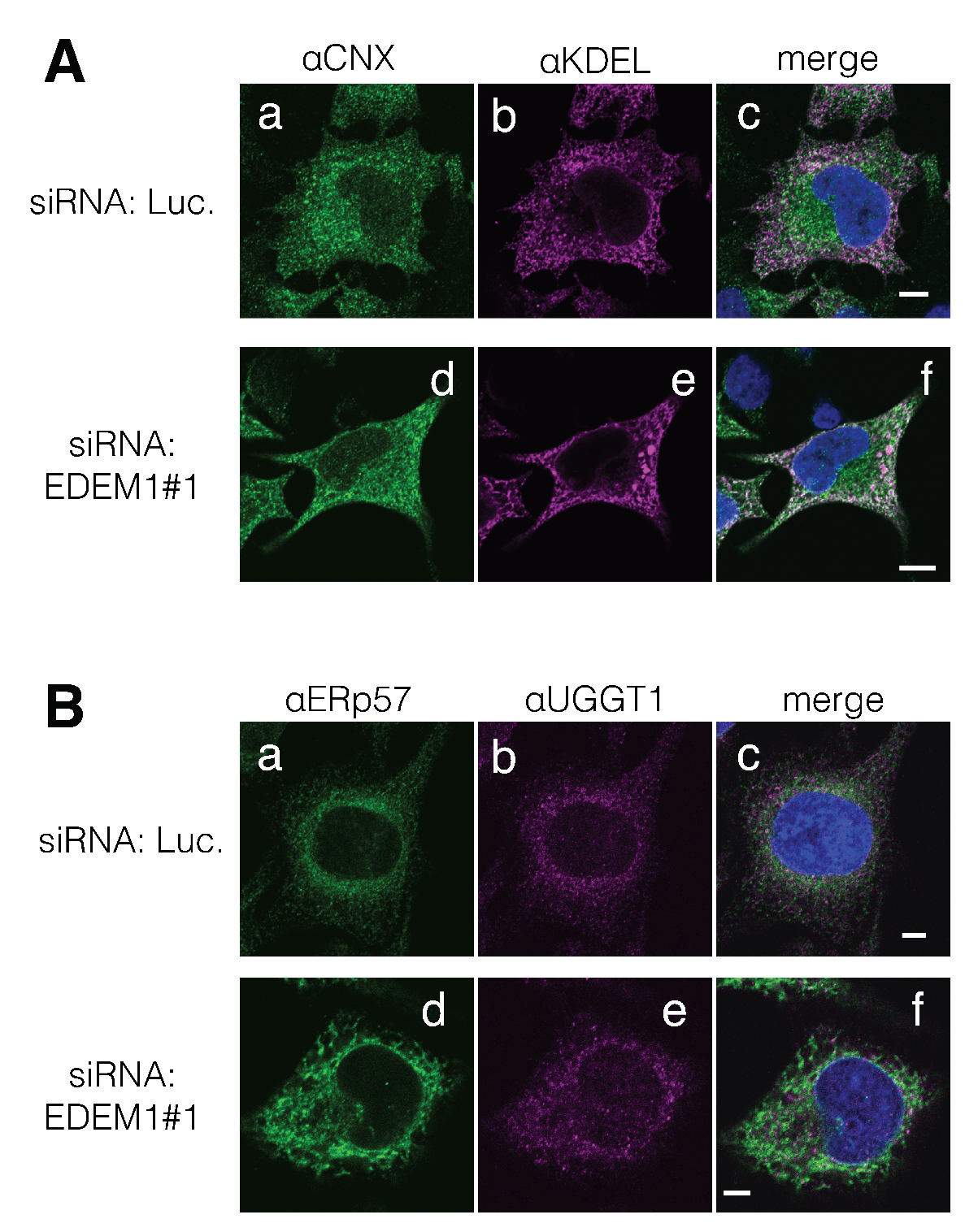


**Figure S2. Knockdown of EDEM1 did not result in accumulation of CNX and UGGT1**

HeLa cells were incubated with siRNA for negative control (luciferase, Luc.) or EDEM1#1 for 72 h. After fixation, indirect immunostaining using anti-CNX and anti-KDEL (**A**) or anti-ERp57 and anti-UGGT1 (**B**) antibodies was conducted. Anti-CNX and anti-ERp57 were visualized with Alexa Fluor 488 (green), while anti-KDEL and anti-UGGT1 were visualized with Alexa Fluor 594 (magenta). Images were obtained using confocal laser microscopy (LSM780; Zeiss). Merged images after DAPI staining and scale bars (represent 10 µm) are shown in panels c and f.


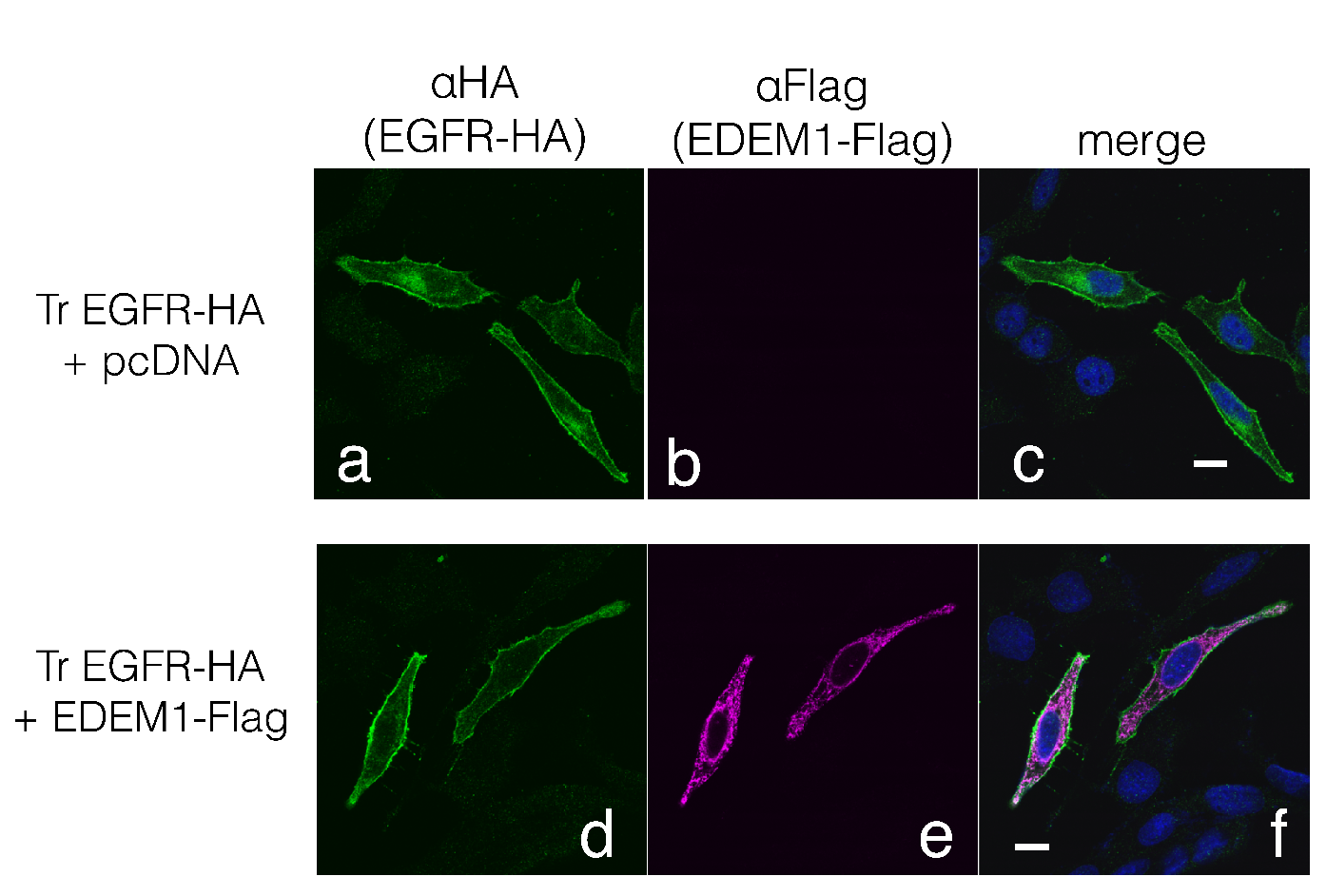


**Figure S3. Cellular trafficking of EGFR-HA co-expressed with EDEM1-Flag**

Immunocytochemistry of HeLa cells transfected with EGFR-HA with pcDNA (empty vector, upper panel) or EDEM1-Flag (bottom panel) for 48 h was performed. Anti-HA for EGFR-HA (a and d) was visualized with Alexa Fluor 488 (green). Anti-Flag for EDEM1-Flag (b and e) was visualized with Alexa Fluor 594 (magenta). Images were obtained using confocal laser microscopy (LSM780; Zeiss). Merged images with DAPI staining and scale bars (represent 10 µm) are shown in panels c and f.


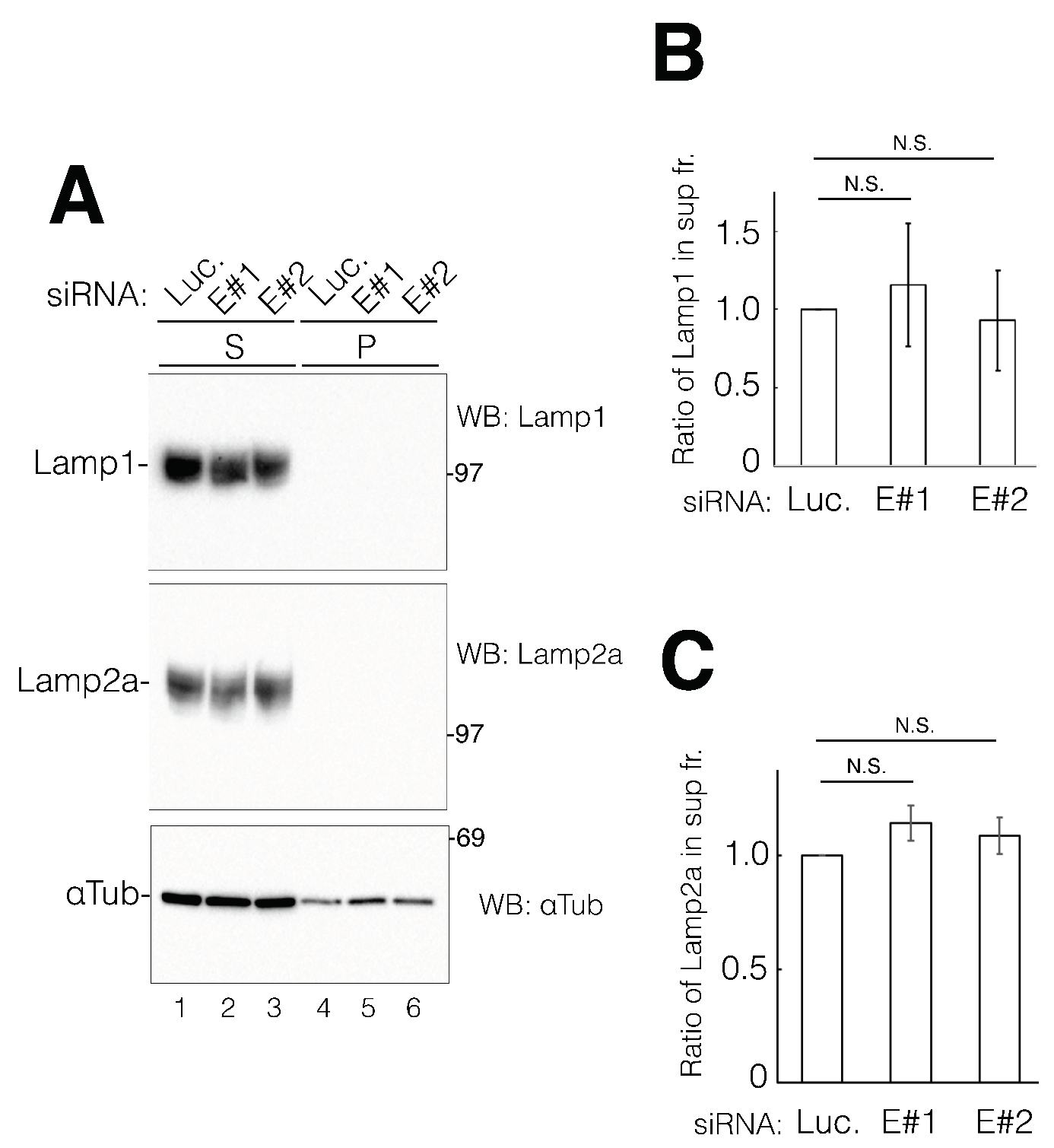


**Figure S4. Solubility of Lamp1 and Lamp2a was not affected by knockdown of EDEM1**

HeLa cells were treated with siRNA for control or EDEM1 (Figure 4A). After the separation of the cell lysate into supernatant (S) and precipitation (P) fractions, Lamp1 and Lamp2a (**A**) were detected by western blotting with indicated antibodies. Each corresponding band was quantified, and the levels were normalized to those of αTubulin (**B, Lamp1**. **C, Lamp2a**). Asterisks refer to the control sample, indicating statistical significance (N.S., not significance) by Student’s *t-*test. Data represent the mean ± standard deviation of three independent experiments.
