# Supplementary material for: Identification of EGF Receptor and Thrombospondin-1 as Endogenous Targets of ER-Associated Degradation Enhancer EDEM1 in HeLa Cells
Source: Int J Mol Sci. 2023 Jul 29;24(15):12171. doi: 10.3390/ijms241512171 (PMC10418772; doi:10.3390/ijms241512171)
Supplement: Supplementary file 1 [file ijms-24-12171-s001.zip › Table S1.pdf]

Table S1. Sequence of primers and siRNAs used in this study

name	sequence (5'-3')
GAPDH RT	CTCTTCCTCTTGTGCTCTTGC
GAPDH r	CCATGAGGTCCACCACCC
GAPDH f	CCACAGTCCATGCCATCAC
EDEM1 r	CTGCAGTCCAGGGAAAAAG
EDEM1 f	GTGGAATTTGGGATTCTGAGTC
EDEM1RT	CATTTCAGACTGGACTTTGGC
XBP1 RT	GAAGTACAGACAGGCTTCTCTGCTATC
XBP1s f	GTGCGTAGTCGACAGCTATGGTGGTGGTG
XBP1s r	GGATCATTCCTCGAGCACTAATCAGCTGGG
Luciferase sense	CGUACGCGGAAUACUUCGAdTdT
Luciferase antisense	UCGAAGUAUUCCGCGUACGdTdG
EDEM1 #1 antisense	UUUCACUGCUCGUCUGGCCdTdT
EDEM1 #1 sense	GGCCAGACGAGCAGUGAAAdTdT
EDEM1 #2 antisense	UUGGAGUCAGUUAUUAUUCdTdT
EDEM1 #2 sense	GAAUAAUAACUGACUCCAAdTdT
EGFR-HA f	ACCGAGATCTCTCGAATGCGACCCTCCGGGACGGC
EGFR-HA r	TGGGTAGCCGGTACCTGCTCCAATAAATTCACTGCTTTG
EGFR-HA RT	CTAAGAGCTAATGCGGGCATGGCTG
EDEM1-Flag NotI f	CTTTAAAATAATGCCCGCGGCCGCCGC
EDEM1Flag HpaI r	CCCGGGTGGCATCCCTGTTAACCCTCC
